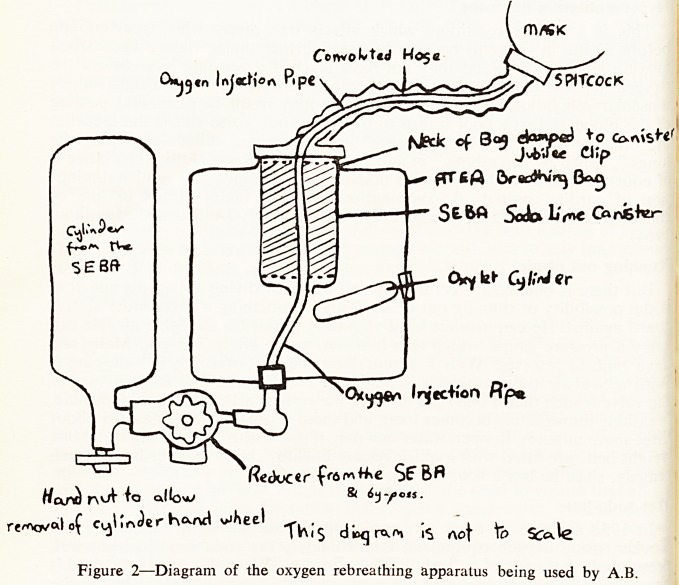# Cave Diving Hazards

**Published:** 1967-04

**Authors:** Oliver C. Lloyd


### CAVE DIVING HAZARDS

BY

OLIVER C. LLOYD

*In this address, which was given to the Bath Clinical Society on November 26th, 1966, the author describes some of the difficulties that may beset a cave diver, more particularly those that may be eliminated by training. He then makes a study of three fatalities among cave divers, in order to see what lessons may be drawn from them, and concludes by setting forth some of the more recent ideas on the subject of drowning.*

### THE PROBLEMS

The first problem of the diver arises from the fact that air is compressible. An extreme example is the pearl diver. At a depth of 33 feet he is under a pressure of two atmospheres, so that the air in his chest takes up half the space. At 66 feet it takes up one third and at 100 feet only one quarter of the space it occupied at the surface. This is equal to the " residual volume ", which is the amount of air left in the chest after forcible expiration. This fact sets a limit to the depth of his dive, for should he dive deeper the negative pressure in his chest would either crack his ribs, or suck fluid out of his pulmonary vessels, so that he would develop pulmonary œdema. Among divers this is known as " the squeeze ". The pearl diver is a " free diver ", that is, he dives without breathing apparatus. In training, a cave diver will practice free diving with a snorkel and face mask, but generally not below a depth of 20 to 30 feet.

### Buoyancy

If you fill your lungs with air at the surface and dive, you are very buoyant until about 15 feet deep, where you reach equilibrium. Below this the buoyancy is negative and you begin to sink. This is simply our old friend Archimedes and his principle. Compressed air displaces less water. With breathing apparatus consisting of a compressed air bottle and a valve, the loss of buoyancy is less marked, but is still present. The valve delivers air at the ambient pressure, so that the lungs can be filled and emptied without effort. Their displacement is the same as at the surface. This is not true of the air in the stomach and intestines. Compression of these organs at depth results in loss of buoyancy. Moreover a cave diver will usually wear a " wet suit " to keep out the cold. This is a suit made of foam neoprene, tailored to be skin-tight. It will admit water, but in such small quantities that it soon warms up, and the absorbed water does not easily exchange with the surrounding cold water. Thus the foam neoprene has excellent insulating properties. It is very buoyant because of the minute air cells in the fabric. The buoyancy of a wet suit is equal to about 16 lbs.; but of course at 33 feet its buoyancy is only 8 lbs. and so on. Hence the diver feels himself getting heavier the deeper he goes, and that means he has to do more work, and so he uses up more air.

You might think that on returning to the surface the air in the stomach and intestines would return to its previous volume. This is often not so, because of ærophagy. This is not simply swallowing air; it is a condition in which air is sucked into the stomach through a relaxed œsophagus. Some say it is due to anxiety. I think not. One needs a little air in the stomach for comfort, and if this is reduced in volume by compression at depth one sucks in a little more. The result is that on return to the surface the stomach is distended, which is very uncomfortable and the discomfort can only be relieved by belching. Now some people are much better at belching than others, and I have met trainees who have continued to feel uncomfortable, simply because they could not belch.

**Free Ascent**

A free ascent from depth is fraught with a much greater danger, namely the risk of burst lung, with possible hæmoptysis, interstitial emphysema, pneumothorax, or even air embolism. This does not happen to the pearl diver, because he finishes with as much air in his chest as he started with. But if you take a breath from a bottle and valve at, say, 20 feet, and then hold your breath while you ascend, the lungs quickly get over-distended. The trainee has to be warned to breathe out all the time he is coming up. It is rather astonishing to learn that damage can be done by free ascent from only 6 feet of water with the breath held. A friend of mine was playing a game of " chickens " with some others. They had a breathing apparatus on the bottom of a swimming bath and took it in turns to take a breath from it, seeing who could hold out the longest before taking another breath. My friend couldn't wait his turn and surfaced rapidly without breathing out. He got an acute pain in his chest and a slight hæmoptysis. Fortunately he recovered completely and he is now one of the best cave divers in the country. The trouble is that, owing to the lack of facilities, so many of us have had to train ourselves. This is a slower process than with proper training and may be a risky one. A simple calculation will show that during an ascent from some depth the lungs get more distended, for each six feet you ascend, the nearer you are to the surface.

**Air in the head**

The middle ears and accessory nasal sinuses are rather a special problem. Unlike the stomach but like the lungs they fill with air at the ambient pressure. If they fail to do so, even for a moment, the subject experiences sharp pain, since they are cavities that cannot collapse under pressure, as can the stomach. Some people can clear their ears and sinuses without conscious effort. They are lucky. Others, like myself, have to be thinking of them all the time. The easiest way to clear the ears is to make them click by swallowing. This can be done quite easily even with a face mask and gag in place, but it needs a little practice. The sinuses may be more difficult. My right frontal sinus is the troublesome one. At a depth of about 10 feet it begins to ache. The thing to do is to squeeze one's nostrils with thumb and forefinger and blow. This forces air into the sinus with a noise like a cat me-owing, and immediately the pain goes.

When the nose is congested, as with a cold with sinusitis, it may be impossible to clear the sinuses or even the ears. In these circumstances one has to forego one's diving. But even then science may come to the rescue, and one may effect decongestion by the use of a Benzedrex inhaler. The effect, however, wears off after about an hour. Oddly enough it is only in descending that one has to worry about clearing the ears or sinuses. On re-ascending it seems that air is able to leave them and enter the nose and naso-pharynx without any trouble. There seems to be some valvular action of the mucous membrance of the tubes which lead to them.

It is far easier to clear the ears and sinuses in an upright position, than when one is upside down. Now this is rather a nuisance, because a diver must train to feel equally comfortable in any position. I think the explanation is this. Everyone knows how posture affects the degree of nasal congestion. If you have a cold and lie on your right side, the left nostril will soon clear itself and allow an airway. The process is too rapid to be accounted for by the absorption of œdema fluid, and I'm fairly sure it is simply due to alteration in the contents of the blood vessels. The nasal mucosa, particularly that of the turbinates and septum, contains a rich plexus of venous sinuses, which are filled and emptied by the action of the smooth muscle in their walls. When you are upside down, the mucosa around the orifices of the frontal sinuses is more congested, and when you turn the right way up it can become decongested and allow air to enter the sinuses.

**Ear drums**

Failure to clear the ears can result in more than mere discomfort, it can be painful. Worse, it can burst the ear drum. The cautious diver will descend very slowly if he has any difficulty in clearing his ears, and will return to the surface if he finds that he is getting pain in the ear unrelieved by swallowing or nose-blowing. The mucous membrane of the Eustachian tube is rather easily damaged by failing to clear the ears. The resulting hæmorrhage may be very slight, but can be enough to prevent the ear from clearing properly for the next day or so. But the cave diver is in a much more difficult position if, having passed through a deep syphon, he gets ear pain on the return. This happened to two of my companions in Wookey Hole on one occasion. One of them, diving upstream from the 9th Chamber, descended 75 feet to the 15th and then up to the surface in the 18th Chamber. There was hardly any surface there, so he fixed his line reel and came back. On his return he experienced acute pain in one ear but was obliged to persist with his dive, in order to regain the 9th Chamber. A second diver then followed exactly the same course as the first, had trouble with tangled lines in the 18th Chamber, returned in a slight hurry, more than slightly anxious, and got pain in his ear in exactly the same way as the first. My turn came next and I " chickened out ". These two divers were lucky not to have got burst drums, which has happened to another diver I know.

I have mentioned the effect of " squeeze " on the chest of a free diver. Squeeze may also affect the eyes and external ears of a bottle diver, if he is not careful. If goggles are worn when diving (as they never should be) the air imprisoned behind them acts like a partial vacuum, resulting in conjunctival hæmorrhage. The reason why the face mask is designed to include the nose is to allow air to enter this space from the nose, so that the pressure behind the mask can be kept equal with that of the water outside. The diver should not expect this equalization to take place without conscious effort, otherwise he may still get a bloodshot eye.

Similarly, if ear plugs are worn to keep the ears dry (as they never should be), they may get driven into the external ear by the pressure at depth, and cause damage. Damage can also be produced if the hood fits too tightly over the ear, so as to produce an airtight seal over the external ear. The damage which can result from this is properly called " barotrauma ", but is often known as " reversed ear ". What happens is that a negative pressure develops in the external ear during descent, and this results in small haemorrhages under the skin surface. Hæmorrhagic blebs may form and these may burst. The damage is not to the ear drum, which is rather odd; this membrane is very sensitive to pressure inwards, but may be forced outwards quite a lot without damage. But the skin of the external auditory tube is bound down to the bone and cartilage and cannot yield to the negative pressure. So it gets damaged first. Acute barotrauma is not an uncommon lesion among professional divers, generally occurring at a depth of between 30 and 50 feet. Jarrett (1961) gives the incidence as once in 200 dives. It can easily be prevented by making sure that air can be blown under the hood from the face mask through the nose.

Quite commonly nose-bleed is encountered among divers who have been having difficulty with ear or nose clearing. It usually causes no real trouble. More troublesome is the tooth-ache, suffered by some trainees who have unfilled holes in their teeth. At the base of a carious lesion air may get trapped quite close to the pulp. Diving will then cause a partial vacuum and affect the nerve of the pulp. The pain may persist for some time.

Normally the pressure in the outer ear is equalized at depth by the entry of water. This is quite harmless, as long as the drum is intact. Water should on no account be allowed to enter the middle ear, as it usually sets up troublesome inflammation. A perforated ear drum is one of the few absolute contra-indications to diving in an otherwise healthy individual.

### Overbreathing

A diver is careful to conserve his air as much as possible. To this end he consciously controls his respiration rate. Most divers can feel comfortable taking one deep breath, and holding it slightly, every eight seconds. If however you breathe in the same way at a depth of 33 feet, you will use up twice as much air. This is wasteful, because the actual oxygen needs of the tissues for respiration are the same at any depth. In theory therefore a breath ought to last much longer the deeper you dive. This is only true up to a point, because bad mixing in the pulmonary alveoli may prevent the available oxygen from being used. Several factors tend to make a diver overbreathe. Some divers do it the moment they get a gag into their mouths. These may not become good divers. Most divers overbreathe when they get into cold water; that is something very difficult to correct. Extra effort may cause overbreathing, and this can be corrected by relaxation. But the worst bugbear is anxiety, and this can only be overcome by proper training. The first time I went diving upstream from the 3rd Chamber in Wookey Hole (Fig. 1) I was in the company of a very experienced member of the Cave Diving Group. He had a large electric light bulb in his hat, which shone beautifully. Unfortunately it got rather hot and burst just as we reached the bottom of the 8th Chamber. While he was getting out his hand-torch the mud got so thick that visibility was almost nil. He then disappeared on the line up to the 9th Chamber. Why I was unable to find this line (it was attached to the brick I was holding) I do not know; but I couldn't. So I sat there trying to feel relaxed. Looking at myself I saw a slightly frightened caver vigorously overbreathing. So I said to him " Lloyd, don't be a fool. If you can't go forward, go back ". So he did that, easily following the line home.

Figure 1—Plan and Section of Wookey Hole (from " The Mendips ", by A. W. Coysh, E. J. Mason and V. Waite, p. 58).

I mentioned cold water as a cause of overbreathing. It should not generally be a serious cave diving hazard, because the water that enters a wet suit quickly gets warmed by the body. On the other hand, one has to remember that the insulating properties of a wet suit become reduced at depth, because of compression of the air cells in the foam neoprene. To wear a double thickness of foam neoprene is undoubtedly warmer, but it does increase the buoyancy problems. Fortunately cave dives are not usually long enough for one to get miserably cold. An efficient hood is essential, as cooling of the back of the neck can cause loss of balance and direction.

**Decompression sickness**

This is a serious hazard for naval and professional divers, but does not affect the cave diver, unless he does repeated dives in one day with a series of bottles. This is because he is not able to carry enough air with him to stay long enough at depth. For the same reason nitrogen narcosis is not a hazard.

**Oxygen equipment**

Most cave diving nowadays is done with open circuit compressed air, so that the hazards of using closed circuit oxygen apparatus are no longer with us. Up until about 1962 this was not so, and all the early exploration by the Cave Diving Group was with closed circuit oxygen The carbon dioxide is absorbed by soda lime, a constant flow rate of oxygen is used and one breathes in and out of a bag called a " counter lung ". Such equipment cannot safely be used below a depth of 30 feet. At higher pressures than two atmospheres the partial pressure of oxygen in the tissues reaches toxic levels and interferes with the respiratory enzymes of the cells. Convulsions occur without warning and the results can be fatal. Another difficulty is the need to ensure that all the nitrogen is expelled from the system before going under water. This is done by carrying out a breathing drill. Failure to do it may result in all the oxygen being used up without warning, since there may be enough nitrogen in the counterlung to prevent " clipping ". (" Clipping " is the feeling experienced when one cannot fill one's lungs, because there is not enough air in the counterlung.) Another trouble may be carbon dioxide excess, either through excessive bodily activity or from some defect in the resorption mechanism in the soda-lime canister. This can result in over-breathing, extreme discomfort, and blackout. Any form of blackout is likely to be fatal for a diver, since he may lose his gag and inhale water. It is only with the full face mask that this may be avoided.

**Shallow water blackout**

This is a form of syncope that may affect divers using oxygen. Recently Captain Miles (1962) has shown that it is due to a combination of factors of which the most important are hyperventilation, assuming an erect position, and performing the Valsalva manœuvre. Unfortunately syncope under water is very likely to be fatal, so that the condition becomes important. Miles showed that if a subject assumes a horizontal position and overbreathes and then assumes an upright position, syncope may take place if it is oxygen that is being breathed but not if it is air. The manœuvre necessary to clear nasal sinuses is very similar to the Valsalva manœuvre, and it is therefore of great importance not to do it after overbreathing. Shallow water blackout can be eliminated by using air sets instead of oxygen.

### Hyperventilation blackout

This is a similar condition, which affects free divers who hyperventilate before diving in order to be able to swim further under water. The carbon dioxide level in the blood may not have reached a warning level before the oxygen tension has fallen to a dangerously low level. Here again, sudden muscular relaxation at the end of the dive may result in peripheral pooling of the blood and so a poor return to the heart; but worse still is the fact that the breath holding can be prolonged by a manœuvre called " pumping the lungs ", which is " breathing " in and out against a closed glottis. The latter is of course the same as the Valsalva manœuvre and results in a sudden diminution of cardiac output. A combination of these factors leads to loss of consciousness, often fatal (Craig, 1961; B.M.J., 1961; Dmitru and Hamilton, 1964).

### Running out of air

But there is one hazard common to all forms of diving apparatus and that is the possibility of running out of air. This is something a diver must always guard against. He can arrange his diving time to suit his available air. He can carry a pressure gauge, which tells him how much air he has got. Many sets have built-in reserves. With a counterlung the first warning is " clipping ". With a Scubair the warning is difficulty in sucking air. The construction of the valve is such that if one rises towards the surface after getting this warning, breathing immediately becomes freer, and there is usually enough air for about three more minutes. In open water one may if one wishes surface rapidly. Most weight belts are fitted with a quick release buckle. The cave diver has no such remedy, since he has a roof of rock over his head.

### Wet soda-lime

In 1956 a friend of mine was exploring the second sump in Stoke Lane Slocker, using oxygen equipment. Unfortunately the soda lime had got wet, presumbaly because of a leak in the canister. When he came to do his breathing drill before diving he got a mouth full of caustic soda, which quite upset him. To add to his troubles the only water available for mouth washing was the sump water, which is about the dirtiest imaginable. Nevertheless that was what he used.

### THREE FATALITIES

A study of three fatal accidents to cave divers will emphasize the nature of some of the more serious hazards.

The first concerns A.B., a man aged 30. On 3.11.62 he was trying out a new, home-made oxygen rebreathing apparatus in the Mineries Pool at Priddy. The diagram (Fig. 2) shows its construction. It should be noted that the reducing valve from the SEBA cylinder was originally designed to give a flow rate of three litres a minute. For oxygen this is too much, and the screw setting had been adjusted to give an estimated flow of about one litre a minute. Unfortunately the flow rate at this speed is not constant and may vary between 1 litre per minute and nothing at all. Another criticism of the design concerned the soda-lime canister. This was not designed for continuous underwater swimming, being of too small a capacity and cross-section. The soda-lime was of good quality but the grains were coarser than they should be. The central inlet tube would cause differential cooling of the soda-lime and

Figure 2—Diagram of the oxygen rebreathing apparatus being used by A.B.

enhance channelling. All these things would tend to give a $CO_2$ build-up on exertion. This is probably what happened.

Too late A.B. tried to get rid of his weight belt, which was not of a quick release type, before losing consciousness. The water was dark and muddy and it was a full hour before he was rescued. During this time the reducing valve was shutting down and giving him only just enough oxygen to support life. The anoxia from which he was suffering caused acute pulmonary œdema. Resuscitation by the mouth-to-mouth method was successful but satisfactory spontaneous breathing did not return. He was taken to a local hospital but died there about a quarter of an hour after admission. An emergency such as this requires exceptional facilities for treatment and unusual imagination by those in charge.

Two lessons are learnt. One is the danger of using home-made apparatus composed of bits and pieces originally intended for other purposes. The other is the value of a full face mask. This undoubtedly saved A.B. from drowning.

The second case was that of C.D., a man of 24 years and a keen aqua-lung diver. He was attending a meet of his particular diving club in the old mine shaft of Deep Ecton Copper Mine. About 100 feet down one shaft there was believed to be a side passage, which joined a second shaft 15 feet away. After some of the others had dived C.D. borrowed a twin set air lung which had already been used to a depth of 100 feet or more, so that one of the bottles of air had been used up. It was estimated by members of the party that the remaining air would last for 10 minutes at 100 feet, which was perhaps a bit over-optimistic.

The signalling system arranged was different from the standard code and was unduly complicated. C.D. went down about 100 feet, and signalled that he proposed to turn into the side passage. Next he gave two pulls, which by the standard code means " up ", but by the arranged code meant " pay out more line ". They let him have 240 feet of rope, so that he went very deep, rapidly used all his air and got squeezed. He also got stuck, and the stand-by diver had to go down 100 feet before a big heave on the line freed the subject. When he was pulled up, the bottles were empty, the gag was behind his neck, and he was dead. He, too, was found to have acute pulmonary œdema, just like A.B. It is probable that no water entered his lungs from outside, so he didn't actually drown. He had taken off his weight belt, but this had not given him enough buoyancy to rise.

The lesson is that although air diving is much safer than oxygen, it too has its dangers. At this depth, of course, oxygen diving would have been impossible. The two main faults were: not allowing 100% safety margin for air, and having a perverse signalling system. No conclusion can be deduced from the fact that the gag was not in his mouth, but the next case will show how important it is to be able to replace the gag, if it comes adrift accidentally.

The third case was that of E.F., aged 33, an experienced diver who trained regularly and was an excellent caver. On Easter Sunday of 1964 he was engaged in an operation in Lancaster Hole. The sump at the lower end of the master cave is a pool about 80 feet long with a downward sloping floor. At the end of the operation someone was pulling in the shot-line that had been used, when it stuck about 25 feet from base in about 10 feet of water. E.F. went out to free the line, but in so doing he got tangled up in it, lost his gag, presumably panicked and drowned very quickly. The stand-by diver went after him as soon as the bubble pattern on the surface indicated that the gag had been lost. He freed E.F. within about two minutes and artificial respiration was applied by the mouth-to-mouth method. After another 20 minutes external cardiac massage was added. These were kept up for over two hours but without avail.

On going into the case afterwards it did appear that there were some minor defects in the design of the apparatus. For one thing a cave diver carries his bottle to one side, and therefore a twin hose to the gag is unsuitable. For another, a neck retaining strap to the gag is essential, to prevent its getting altogether lost. But two lessons above all stand out from this incident. One is the great respect which one must have for loose lines in the water. It is not unusual for a line to get caught in one's apparatus or round one's ankle, and one must always stop and carefully undo it. The other is the importance of training all cave divers in resuscitation technique.

### DROWNING AND "NEAR-DROWNING"

A great deal of gloom was cast over the prognosis of subjects recovering from fresh water drowning by Donald (1955), who showed that in the experimental animal fresh water was absorbed very rapidly from the lungs into the blood stream. There it caused hæmodilution, hæmolysis, and electrolytic disturbances well calculated to bring about ventricular fibrillation, from which the animal usually died within a few minutes. He remarked on the paucity of reports in the medical literature of human cases of "near-drowning" in which, after resuscitation, the above trouble from inhaled water had developed. He concluded that such cases did not exist, the sinister interpretation being that inhalation of moderate quantities of fresh water was invariably fatal. This is now known not to be true. A lot has been learnt in the last ten years, and a most useful report on cases of near-drowning has been published by Fuller (1963). He studied 77 cases of near-drowning, besides 20 delayed drowning deaths. His most significant finding was that, despite what happens in the experimental animals, dilution of the blood in man is so quickly corrected that disturbances in salt concentration and hæmolysis are not serious problems. The really serious problems are three: the effects of oxygen deprivation on the brain, pulmonary œdema, and pneumonia due to the inspiration of mud, sand, or vomit.

Oxygen deprivation is directly related to the duration of immersion until resuscitation is started. It is often said that you only have from one to five minutes to save the subject. This is very variable. The most startling late success was reported by Kvittingen and Næss (1963). A boy of five years was submerged in an ice-cold river for 22 minutes; it took him $2\frac{1}{2}$ months to recover. This case is exceptional, and it is true that it is not just minutes that count but seconds.

No time should ever be wasted in trying to tip water out of the throat. Ruben and Ruben (1962) demonstrated this by filling the lungs of dead subjects with measured quantities of fluid. They then tried to see how much of this fluid they could recover by postural drainage or external pressure, and found that the quantity was not significant.

It has now been conclusively shown that the only effective and economical way of giving artificial respiration is by the mouth-to-mouth (or better, mouth-to-nose) method. All the Schæffers and Sylvesters and Holger-Niellsons are completely out of date. They provide far less pulmonary ventilation at the expense of far more effort. But in drowning cases it is often necessary to combine this with external cardiac massage. It is often said that the heart goes on beating after respiration has stopped, particularly in salt water drowning. This is uncertain. Neither is it certain why the heart stops, though it is probably not nearly as often because of ventricular fibrillation as the experimental data would suggest. After giving the subject six breaths, therefore, one should feel for the pulse and look at the pupils. If the pulse is absent and the pupils are dilated, one must start rhymical compression of the heart at the rate of once a second by pressing on the lower end of the sternum. (The method cannot be adequately described and must be taught.) This is not just a form of tittilation of the heart to encourage it to start up on its own; it is an effective method of maintaining the circulation and ensuring that adequately oxygenated blood reaches the brain (Nixon, 1961). In a subject likely to recover, therefore, the pupils will be seen to contract.

In theory, resuscitation by these two methods can be kept up indefinitely. In fact they are not likely to be successful if spontaneous circulation and respiration have not returned in half an hour. It is important to get the subject to hospital as quickly as possible, because more efficient methods of resuscitation are there to be had. Pulmonary œdema may be combatted by giving oxygen at two atmospheres pressure, and defibrillation may often help the heart to recover a normal rhythm. Without these adjuncts the restoration of the heart beat is a distinctly chancy affair.

It is sometimes believed that there is a thing called "dry drowning" where spasm of the larynx stops water from entering the lungs and the subject passes out from asphyxia. There is some doubt whether this occurs. Water in the lungs, especially fresh water, is rapidly absorbed into the blood stream, so that at *post mortem* the lungs may appear dry in any case. Yet in fatal cases of drowning it is usually possible to find traces of inhaled mud in the lungs. On the other hand, there are anomalous cases. I saw recently a man drowned in the Feeder Canal in Bristol. I could find no mud in sections of his lungs, which is surprising, since this canal contains some of the dirtiest water in Bristol. It could be deduced that a significant amount of water did not enter his lungs, and that he died from asphyxia. This is really what is meant by "dry drowning". Thus Fuller may not be right in attributing absence of hæmolysis and disturbance in salt concentration to rapid correction of the hæmodilution. Dry drowning would provide an alternative explanation.

Pulmonary œdema is likely to occur in fresh water drowning just as it is in sea water. In both instances the principal cause seems to be the washing out from the alveoli of pulmonary surfactant. This is a lipoprotein elaborated by some of the alveolar epithelial cells, which has the property of diminishing the surface tension and counteracting the elastic recoil of the lung tissue. It has recently been shown in the Department of Pathology at the University of Bristol (Manktelow and Hunt, 1967) that surfactant is lost in many forms of pulmonary œdema, including that found in cases of recent drowning. The pink foam in the air passages in sea water drowning contains a great deal of pulmonary surfactant.

The inspiration of mud or vomit is usually the cause of further troubles, if resuscitation is successful and pulmonary œdema is surmounted. It is responsible for a delayed pneumonia, which is often severe and fatal after a day or two.

It is difficult to find out what proportion of drowning cases are saved, but there are some indications that modern methods of resuscitation are useful. In 1963 life-guards at Biarritz were taught to combine external cardiac massage with mouth-to-mouth respiration in cases of drowning (Genaud, Legendre and Saury, 1965). During the next two years they dealt with 46 incidents, excluding the hopeless cases. 32 subjects were still breathing and their hearts were still beating; these all recovered without trouble. There was recent cardiac arrest in 14 cases, and two of these subjects recovered with external cardiac massage. This is not a high proportion of successes, but it is encouraging. In sum we study methods of resuscitation and use them when need arises, not only because we must, but with some degree of confidence, because we know that there is some chance of their success.

REFERENCES

*Brit. Med. Jl.* (1961) (leading article), **i** 1596.
Craig, A. B. (1961) *Jl. Amer. med Ass.*, **176,** 255.
Dmitro, A. P. and Hamilton, F. G., (1964) *Amer. Acad. gen. Practice*, 123-125.
Donald, K. W. (1955) *Brit. med. Jl.*, **ii**, 155-160.
Fuller, R. H. (1963), *Proc. Roy. Soc. Med.*, **56**, 33-38.
Genaud, P. E. M., Legendre, R. and Saury, A. (1965), *Lancet*, **i**, 1383. ...
Jarrett, A. (1961), *Brit. med. Jl.* **ii**, 483-486.
Kvittingen, T. D. and Næss, A (1963) *Brit. med. Jl.*, **i**, 1313-1317.
Manktelow, B. W. and Hunt, A. C. (1967), *Med. Sci. and the Law* (in Press).
Miles, S. (1962), *Underwater Medicine*, Staple Press, p. 129.
Nixon, P. G. F. (1961), *Lancet*, **ii**, 844-846.
Ruben, A., and Ruben, H. (1962), *Lancet*, **i**, 780-781.

## Figures and Tables

**Figure 1 f1:**
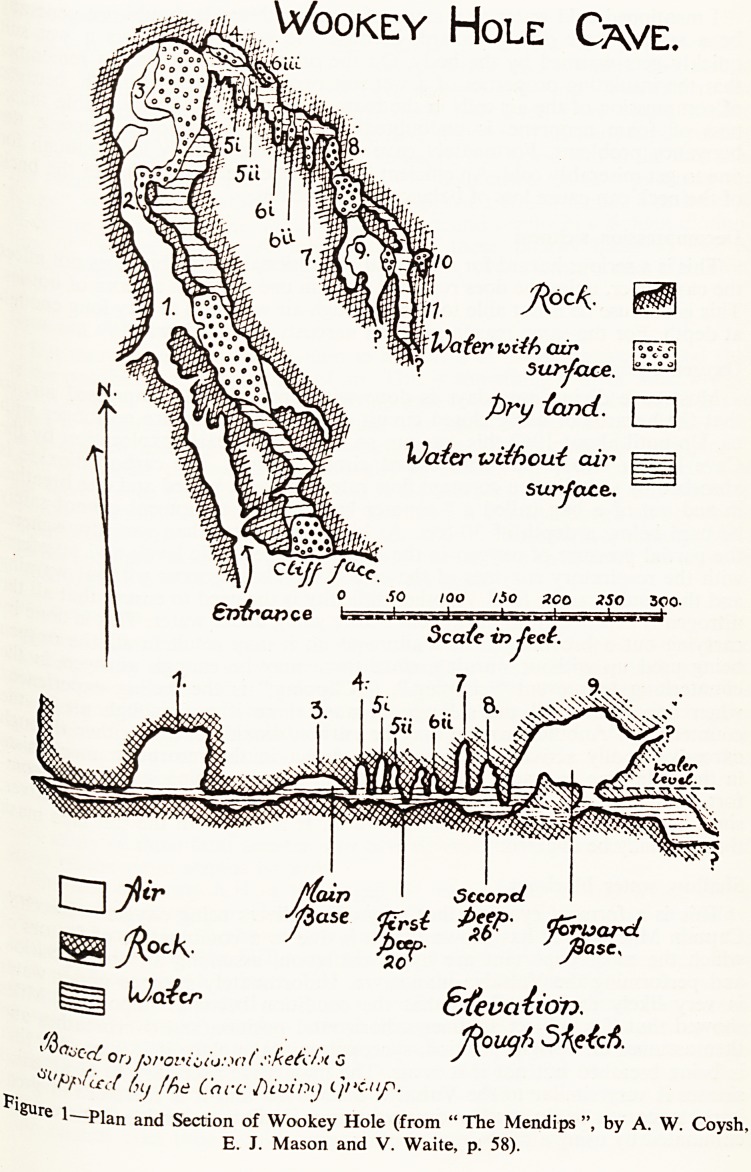


**Figure 2 f2:**